# Prevalence of Postpartum Depression Among Mothers Giving Birth at King Abdulaziz University Hospital, Jeddah, Kingdom of Saudi Arabia From 2020 Until 2022

**DOI:** 10.7759/cureus.31365

**Published:** 2022-11-11

**Authors:** Nada A Alhusaini, Noora A Zarban, Samaa T Shoukry, Maha Alahmadi, Nouf K Gharawi, Rehab Arbaeyan, Badriah A Almehmadi, Wid Kattan, Osama M Bajouh

**Affiliations:** 1 Medical Student, King Abdulaziz University Hospital, Jeddah, SAU; 2 Medical Student, King Abdulaziz University, Jeddah, SAU; 3 Medical Student, King Abdulaziz University Faculty of Medicine, Jeddah, SAU; 4 Division of Psychiatry, Department of Medicine, College of Medicine, King Abdulaziz University Hospital, Jeddah, SAU; 5 Department of Obstetrics and Gynaecology, Infertility, King Abdulaziz University Hospital, Jeddah, SAU

**Keywords:** modifiable risk factors, early screening programs, cross-sectional study, mother-baby relationship, edinburgh postnatal depression scale, depression, postpartum, women health, postpartum depression

## Abstract

Background

Postpartum depression (PPD) is common among women worldwide. PPD affects women after giving birth and can impact the relationship between mothers, their babies, and their spouses, and is associated with considerable economic and personal burdens. This study aimed to calculate the prevalence of PPD among women who gave birth at King Abdulaziz University Hospital (KAUH), Jeddah, Saudi Arabia and identify significantly correlated variables using a cross-sectional approach.

Methodology

This cross-sectional study included a sample size of 483 mothers from the obstetrics inpatient ward at KAUH, Jeddah, Saudi Arabia. Participants were selected randomly and interviewed using a questionnaire consisting of two parts. The first part included demographic data and the studied variables, and the second part was the Edinburgh Postnatal Depression Scale (EPDS). Patients were followed up for six weeks using the same questionnaire in 354 participants.

Results

Of the 483 postpartum women, 15.1% (n = 73) were more likely to exhibit PPD on day one, with a cutoff of 13 points using the EPDS. A smaller proportion of participants were more likely to exhibit PPD at week six (5.1%, n = 18). This finding suggests a positive improvement in the prevalence of PPD from day one to week six. The most significant predictors identified in the regression analysis were family monthly income and family support (p = 0.001 and 0.003, respectively), which positively impacted prevalence.

Conclusions

The calculated PPD prevalence in this cross-sectional study was high. Because PPD negatively impacts the relationship between a mother and her child, with consequences potentially affecting the entire family, we suggest increasing awareness of PPD and focusing on the most significant modifiable risk factors. In addition, early screening programs and continuous follow-up are recommended to provide early intervention and support, which may decrease the harmful impacts of PPD.

## Introduction

According to the World Health Organization (WHO) [1], depression is a widespread mental disorder characterized by persistent sadness and a lack of interest or pleasure in previously rewarding or enjoyable activities that can affect all aspects of life. One of the most common types of depression in women worldwide is postpartum depression (PPD) [2]. Perinatal mental illnesses, such as PPD and anxiety, affect one in six women and are associated with considerable economic and personal burdens [3]. PPD affects some mothers after childbirth and can have a considerable impact on mother-baby relationships [4].

Physical and emotional factors such as unwanted pregnancies, sudden hormonal changes, and feeling overwhelmed by the responsibility of motherhood reportedly play critical roles in the incidence of PPD [5,6]. In addition, mothers with depression may have symptoms ranging from difficulty forming emotional bonds with their babies to feelings of sadness, feeling overwhelmed, anxiety, rage, irritability, and suicidal and homicidal ideation without an apparent cause [5]. PPD symptoms frequently appear within the first few weeks after giving birth. However, the onset may start early during pregnancy or later up to one year after delivery [7].

One of the most effective tools for assessing PPD is the Edinburgh Postnatal Depression Scale (EPDS), which has been used in most research on PPD [8,9]. Different studies have reported varied results regarding the global prevalence of PPD. A previous systematic review and meta-analysis reviewed 291 studies involving 296,284 women from 56 countries and concluded that the global prevalence of PPD was 17.7% [10].

Another study conducted in Iran reported a 25.3% prevalence of PPD among Iranian women [11]. Moreover, some previous studies reported that the prevalence of PPD in some developing countries ranged from 19.8% to 82.1% [12,13].

A study conducted in Riyadh, Saudi Arabia in 2021 reported a PPD prevalence of 38.50% (n = 67) according to EPDS scores [9]. Another study conducted in Jeddah in 2021 using the same scale reported a prevalence of 20.9% [14].

The differences in these results may reflect variability in the actual prevalence among different countries. Indeed, the WHO reports that approximately 10% of pregnant and 13% of postpartum women globally experience a mental disorder, mainly depression. The prevalence is even higher in developing countries, reaching 15.6% during pregnancy and 19.8% after childbirth [12,15].

These numbers indicate that PPD is a significant problem in many communities and is even linked to maternal mortality as suicide is one of the main causes of maternal mortality in the first postpartum year [16,17]. Despite the abundance of literature on PPD, not enough attention has been paid to PPD among women in the Kingdom of Saudi Arabia.

This study aimed to calculate the prevalence of PPD among women who gave birth at King Abdulaziz University Hospital (KAUH), Jeddah, Saudi Arabia between 2020 and 2021 and identify the most significant predictors of prevalence.

## Materials and methods

The Raosoft software (Raosoft, Seattle, WA, USA) was used to calculate the minimum required sample size, which was 345 of 3,360 deliveries in two years [18].

Ethical approval for this study was obtained from the Biomedical Ethics of King Abdulaziz University Hospital (KAUH), Jeddah, Saudi Arabia prior to data collection (approval number: 437-20). The data collection team consisted of fifth- and sixth-year medical students under the supervision of a psychiatrist who specialized in women’s health and an obstetrics and gynecology consultant. Participants were interviewed twice to complete the questionnaire. The first data collection timepoint was immediately after birth using a questionnaire focused on demographic data followed by the EPDS questionnaire. The second data collection timepoint was six weeks postpartum using the EPDS questionnaire only.

The sample initially included 483 mothers from the obstetrics inpatient ward who were followed up at six weeks postpartum. However, we lost contact with 29 participants; thus, the final study sample for our six-week postpartum analysis was 354 participants. The time required to interview each participant was approximately 10 minutes. All participants received the same postpartum care from the hospital staff and were notified of the study objectives and confidentiality policy. Written informed consent was obtained from all participants on the first page of the questionnaire.

As previously mentioned, the questionnaire comprised two parts. The first part included 12 questions regarding demographic data, including age, nationality, marital status, economic status, and employment status, as well as delivery mode, number of children, family psychological support, complications during delivery, medical and psychiatric history, and family history of hereditary disorders.

The second part consisted of a validated Arabic version of the EPDS [8], which was used to screen and identify patients at risk for PPD. The EPDS contains 10 questions that assess a new mother’s emotions over the past seven days before the interview. Every question scored emotions from 0-3. Questions 1, 2, and 4 were scored as 0, 1, 2, and 3, respectively, with the top box scored as 0 and the bottom box scored as 3. Questions 3 and 5-10 were reverse-scored, with the top box scored as 3 and the bottom box scored as 0. The maximum score was 30. A score ≥10 indicated possible depression. Mothers who scored >13 were most likely to develop a depressive illness of varying severity.

However, previous studies have used different cutoff points. A study conducted in Riyadh, Saudi Arabia in 2021 considered a cutoff of 13 points for possible PPD, with scores <13 considered normal [9]. Another study conducted in Iran considered scores ≥13 as PPD, with 10-12 as borderline and 0-9 as no PPD [11]. Furthermore, a study conducted in Jeddah, Saudi Arabia in 2019 reported that a score of <8 points indicates that PPD is unlikely, while 9-11 indicates possible depression, 12-13 suggests a high possibility of PPD, and >13 indicates positive results [14]. Cox and Holden stated that the varying cutoff points in previous studies may be related to differences in population sizes, the timing of EPDS interviews, and differences in culture and expression [19]. In our study, we adopted the same cutoff scores as those used in the Iranian study [11]. The cutoff was 13 points, and scores between 10 and 12 were considered borderline or possible PPD, while scores ≤9 were considered to suggest a lower likelihood of PPD. All participants who stated that they had self-harming thoughts were offered medical assistance.

Statistical analysis

The collected data were analyzed using SPSS version 23 (IBM Corp., Armonk, NY, USA) and GraphPad Prism version 8 (GraphPad Software, Inc., San Diego, CA, USA). Categorical and nominal variables were represented as counts and percentages. On the other hand, continuous variables were represented as means and standard deviations. The chi-square analysis was used to determine relationships between categorical variables, while the one-way analysis of variance was used for comparisons of more than two groups. The tests described above were performed under the assumption of normal distribution. A general linear regression model was used to identify significant predictors of PPD using a main-effect model. A p-value of <0.05 was used to discard the null hypothesis.

## Results

In this study, the prevalence of PPD on day one and at week six after delivery among 483 patients, as well as the predictors influencing PPD during these time points, were evaluated.

The participants had a mean age of 31.58 ± 5.8 (range: 17-53) years, were mostly of Saudi nationality (86.7%, n = 419), delivered naturally (60.2%, n = 192), had a supportive family during pregnancy (89.4%, n = 432), were unemployed or stayed home (78.5%, n = 379), and had enough monthly family income (83.2%, n = 402) (Table 1). All participants were married. Regarding the number of children, nearly half of the participants had one or two children (47.2%, n = 228), 39.5% (n = 191) had three or four children, and a small percentage had at least five children (13.2%, n = 64).

**Table 1:** Sociodemographic characteristics and prevalence of postpartum disorder among the studied population. Min: minimum; Max: maximum; SD: standard deviation; C-section: cesarean section

		Count	%
Total		483	100.0
Nationality	Saudi	419	86.7
	Non-Saudi	64	13.3
Mode of delivery	Natural	291	60.2
	C-section	192	39.8
Number of children	1–2	228	47.2
	3–4	191	39.5
	5–6	47	9.7
	6 and more	17	3.5
Social status	Married	483	100.0
Was your family supportive during this pregnancy?	Yes	432	89.4
	No	51	10.6
Job	Employed	104	21.5
	Unemployed	379	78.5
Family monthly income	Not enough	71	14.7
	Enough	402	83.2
	More than enough	10	2.1
Changes and birth complications	None	175	36.2
	1	125	25.9
	2	108	22.4
	3 or more	75	15.5
Are you known to have a chronic disease?	Yes	75	15.5
	No	408	84.5
Family history of hereditary disorders	Yes	217	44.9
	No	266	55.1
Are you known to have any psychiatric illness?	Yes	9	1.9
	No	474	98.1

At least one cumulative birth complication was observed in most patients (73.8%, n = 308). Among the types of changes and birth complications, stretch marks were the most common, followed by weight gain, obstructed labor, scars, bleeding, and psychological trauma. Most participants denied having a chronic disease (84.5%, n = 408) or psychiatric illness (98.1%, n = 474) and did not have a family history of hereditary disorders (55.1%, n = 266) (see Table 1).

Regarding the potential for self-harm, three (0.6%) patients reported having thoughts “quite often to sometimes” of harming themselves on postpartum day one. In contrast, only one (0.3%) participant reported thinking of self-harm “sometimes” at week six, suggesting an improvement in PPD symptoms over time. With regard to PPD prevalence on day one, the majority of the participants were considered “less likely” to exhibit PPD (71.8%, n = 347), with 13.0% (n = 63) who “possibly” had PPD and 15.1% (n = 73) “most likely” had PPD.

Of the 479 patients who had never thought of harming themselves during this period, nearly three-fourths were less likely to exhibit PPD. The proportion of participants who were less likely to exhibit PPD increased at week six (88.7%, n = 314), and the number of participants who possibly (6.2%, n = 22) and most likely had PPD (5.1%, n = 18) decreased. In addition, of the 351 patients who never thought of harming themselves during this period, more than three-fourths were less likely to exhibit PPD. This finding suggests a positive improvement in the prevalence of PPD from day one to week six.

Table 2 and Table 3 show the association of sociodemographic characteristics and the prevalence of PPD among participants on day one and at week six according to the chi-square test with a <0.05 significance level. The results revealed that family support during pregnancy was significantly associated with PPD (p = 0.003). More specifically, we observed an inverse relationship between PPD and family support, with a significantly higher proportion of patients who were less likely to exhibit PPD on day one (74.1%, n = 320) who received family support during their pregnancies compared with patients who possibly (12.5%, n = 54) and most likely (13.4%, n = 58) had PPD.

**Table 2:** Association of sociodemographic characteristics with PPD prevalence among participants at day one. ^a^significant (<0.05) using the chi-square test. PPD: postpartum depression; C-section: cesarean section

Variables		Total	Day 1			P-value
			Less likely	Possibly	Most likely	
Total		483	347 (71.8%)	63 (13.0%)	73 (15.1%)	-
Age		483	31.63 ± 5.6	30.68 ± 5.8	32.12 ± 6.3	0.333
Nationality	Saudi	419	299 (71.4%)	59 (14.1%)	61 (14.6%)	0.187
	Non-Saudi	64	48 (75.0%)	4 (6.3%)	12 (18.8%)	
Mode of delivery	Natural	291	215 (73.9%)	33 (11.3%)	43 (14.8%)	0.349
	C-section	192	132 (68.8%)	30 (15.6%)	30 (15.6%)	
Number of children	1–2	228	161 (70.6%)	35 (15.4%)	32 (14.0%)	0.602
	3–4	191	139 (72.8%)	19 (9.9%)	33 (17.3%)	
	5–6	47	33 (70.2%)	7 (14.9%)	7 (14.9%)	
	6 and more	17	14 (82.4%)	2 (11.8%)	1 (5.9%)	
Was your family supportive during this pregnancy?	Yes	432	320 (74.1%)	54 (12.5%)	58 (13.4%)	0.003^a^
	No	51	27 (52.9%)	9 (17.6%)	15 (29.4%)	
Job	Employed	104	84 (80.8%)	9 (8.7%)	11 (10.6%)	0.073
	Unemployed	379	263 (69.4%)	54 (14.2%)	62 (16.4%)	
Family monthly income	Not enough	71	42 (59.2%)	12 (16.9%)	17 (23.9%)	0.001^a^
	Enough	402	301 (74.9%)	50 (12.4%)	51 (12.7%)	
	More than enough	10	4 (40.0%)	1 (10.0%)	5 (50.0%)	
Changes and birth complications	None	175	129 (73.7%)	22 (12.6%)	24 (13.7%)	0.233
	1	125	97 (77.6%)	14 (11.2%)	14 (11.2%)	
	2	108	76 (70.4%)	14 (13.0%)	18 (16.7%)	
	3 or more	75	45 (60.0%)	13 (17.3%)	17 (22.7%)	
Are you known to have a chronic disease?	Yes	75	52 (69.3%)	10 (13.3%)	13 (17.3%)	0.830
	No	408	295 (72.3%)	53 (13.0%)	60 (14.7%)	
Family history of hereditary disorders	Yes	217	153 (70.5%)	31 (14.3%)	33 (15.2%)	0.753
	No	266	194 (72.9%)	32 (12.0%)	40 (15.0%)	
Are you known to have any psychiatric illness?	Yes	9	3 (33.3%)	1 (11.1%)	5 (55.6%)	0.003^a^
	No	474	344 (72.6%)	62 (13.1%)	68 (14.3%)	

**Table 3:** Association of sociodemographic characteristics with PPD prevalence among participants at week six. ^a^significant (<0.05) using the chi-square test. PPD: postpartum depression; C-section: cesarean section

Variables		Total	Week 6			P-value
			Less Likely	Possibly	Most Likely	
Total		354	314 (88.7%)	22 (6.2%)	18 (5.1%)	-
Age		354	31.24 ± 5.7	31.55 ± 6.3	32.50 ± 4.7	0.652
Nationality	Saudi	305	271 (88.9%)	19 (6.2%)	15 (4.9%)	0.652 0.938
	Non-Saudi	49	43 (87.8%)	3 (6.1%)	3 (6.1%)	
Mode of delivery	Natural	214	190 (88.8%)	14 (6.5%)	10 (4.7%)	0.872
	C-section	140	124 (88.6%)	8 (5.7%)	8 (5.7%)	
Number of children	1–2	171	150 (87.7%)	13 (7.6%)	8 (4.7%)	0.886
	3–4	141	125 (88.7%)	8 (5.7%)	8 (5.7%)	
	5–6	29	27 (93.1%)	1 (3.4%)	1 (3.4%)	
	6 and more	13	12 (92.3%)	0 (0.0%)	1 (7.7%)	
Was your family supportive during this pregnancy?	Yes	316	285 (90.2%)	18 (5.7%)	13 (4.1%)	0.024^a^
	No	38	29 (76.3%)	4 (10.5%)	5 (13.2%)	
Job	Employed	81	73 (90.1%)	5 (6.2%)	3 (3.7%)	0.811
	Unemployed	273	241 (88.3%)	17 (6.2%)	15 (5.5%)	
Family monthly income	Not enough	52	46 (88.5%)	1 (1.9%)	5 (9.6%)	0.310
	Enough	298	264 (88.6%)	21 (7.0%)	13 (4.4%)	
	More than enough	4	4 (100.0%)	0 (0.0%)	0 (0.0%)	
Changes and birth complications	None	121	107 (88.4%)	10 (8.3%)	4 (3.3%)	0.429
	1	93	84 (90.3%)	5 (5.4%)	4 (4.3%)	
	2	81	74 (91.4%)	3 (3.7%)	4 (4.9%)	
	3 or more	59	49 (83.1%)	4 (6.8%)	6 (10.2%)	
Are you known to have a chronic disease?	Yes	51	48 (94.1%)	1 (2.0%)	2 (3.9%)	0.352
	No	303	266 (87.8%)	21 (6.9%)	16 (5.3%)	
Family history of hereditary disorders	Yes	152	136 (89.5%)	9(5.9%)	7 (4.6%)	0.916
	No	202	178 (88.1%)	13 (6.4%)	11 (5.4%)	
Are you known to have any psychiatric illness?	Yes	4	4 (100.0%)	0 (0.0%)	0 (0.0%)	
	No	350	310 (88.6%)	22 (6.3%)	18 (5.1%)	

Family income was also significantly associated with PPD prevalence (p = 0.001). Specifically, a significantly higher number of participants who were less likely to exhibit PPD on day one (74.9%, n = 402) were receiving enough monthly family income compared with patients who possibly (12.4%, n = 50) and most likely (12.7%, n = 51) had PPD during this period.

A history of psychiatric illness was also significantly associated with PPD prevalence (p = 0.003). In particular, significantly higher proportions of patients who were most likely to exhibit PPD on day one (55.6%, n = 5) had a psychiatric illness. This finding implies that psychiatric illness significantly contributes to an increased risk of developing PPD in the early weeks of pregnancy. No significant association was observed with employment status (p = 0.073).

At week six, PPD prevalence was only significantly associated with family support during pregnancy (p = 0.024). Specifically, significantly more patients who were less likely to exhibit PPD (90.2%, n = 285) received family support during pregnancy compared with patients who possibly (5.7%, n = 18) and most likely (4.1%, n = 13) had PPD at week six.

The predictors of PPD on day one and at week six were then evaluated using a general linear regression model with a significance level of <0.05. Our analysis revealed that having any psychiatric illness was the highest predictor of PPD (B = 6.276, p = 0.001), that is, a psychiatric history was associated with a six-fold increase in the risk of having PPD on day one. The second-best predictor was “enough monthly family income” (B = -4.815, p = 0.005). This suggests that having enough family income causes a four-fold decrease in the risk of depression. The third-best predictor of PPD was family support during pregnancy (B = -2.721, p = 0.001), implying that decreased family support inflicts a two-fold increase in the risk of higher depression scores, potentially leading to PPD. The remaining predictors for PPD on day one included employment (B = -1.472) and at least one change and birth complication (B = -2.142, p = 0.006).

At week six, family support during pregnancy (B = -1.580, p = 0.032) and having one change and birth complication (B = -1.646, p = 0.019) were consistent predictors of PPD. These values imply that decreased family support can lead to an increased risk of higher depression scores.

## Discussion

This cross-sectional study was conducted to measure the prevalence and identify significant predictors of PPD among women who gave birth at KAUH, Jeddah, Saudi Arabia between 2020 and 2022. The EPDS was used to screen for PPD, along with a questionnaire regarding sociodemographic data and possible risk factors. The questionnaire was administered during interviews on day one and at week six postpartum. The prevalence at both time points was 15.1% and 5.1%, respectively, with a cutoff of 13 points on the EPDS. Our results differed from the 20.9% prevalence reported in a previous study conducted in Jeddah in 2021 [14]. In contrast, a study conducted in Riyadh in 2020 reported a prevalence of 38.5% [9]. These differences could be related to regional and sample size variations.

The most significant predictor identified in our study was family monthly income (p = 0.001), followed by family support (p = 0.003). Adequate monthly income had a positive impact on PPD prevalence, as it reduced the risk by 4.8 times compared with patients with lower incomes. This finding is consistent with the results of a 2009 study conducted in Riyadh [9]. Conversely, other studies conducted in Saudi Arabia found no correlation between monthly income and PPD prevalence [14,20,21]. The results regarding family support go hand-in-hand with those reported by Alasoom and Koura, in which an association between PPD and having an unsupportive husband [20] was reported. Previous studies by Alsayed et al. and Al-Ghamdi et al. found family support to be insignificant [14,22]. The regression analysis in our study found that having a supportive family reduced the risk of developing PPD by 2.7 times. This indicates the importance of family and spouse support during pregnancy and the postpartum period, as it can be an overwhelming and sensitive time that requires special attention. In addition, other studies reported a significant association between past psychiatric illness and PPD, which supports our findings (p = 0.003) [20,23].

Because we aimed to measure the prevalence of PPD, our study was conducted over a six-week postpartum period to exceed the “baby blues” period, which commonly includes feeling sad, mood swings, and anxiety for approximately one month after giving birth (four weeks). In addition, our study collected data at two time points, day one and the week six follow-up, to compare the prevalence of PPD over time. The calculated prevalence on day one was 15.1% which trended down to 5.1 by week six. This could represent overlapping cases of baby blues or could be due to the stress of pregnancy and childbirth along with the added responsibilities followed by the adaptation that took place over six weeks, in addition to other possible factors that could help mothers cope with this new life event. Therefore, this study suggests that further investigation of the possible modifiable factors could lead to discoveries that could help mothers with PPD improve or even recover.

In contrast to the study by Al-Riyadh, in which mothers who delivered by cesarean section had a higher prevalence of PPD, we found no significant difference between the prevalence among mothers who had spontaneous vaginal delivery compared with those who delivered by cesarean section [9].

In this study, the calculated prevalence was based on the EPDS which is a screening tool for PPD, and therefore, it cannot be used to confirm the diagnosis. In addition, we detected only three participants on day one and one participant at week six who were admitted for having self-harming thoughts. Our small sample size limited our ability to identify an association between PPD and self-harming ideation. Moreover, owing to the lack of unmarried mothers in our sample, we could not measure the effect of marital status on PPD prevalence. These limitations can be minimized by including participants from different healthcare centers throughout Jeddah and increasing the sample size in future studies to obtain a more representative sample of the community. In addition, the sample size was affected by the coronavirus disease 2019 quarantine during the data collection period. Family support and family income were subjectively assessed during the interview and were found to have a strong correlation with PPD, although it should be noted that participants’ perceptions could be affected by their depressive cognition.

## Conclusions

The prevalence of PPD among the participants of this cross-sectional study was lower than that reported in previous studies. Monthly family income was the most significant predictor (p = 0.001), followed by family support and psychiatric history (p = 0.003). We suggest further investigation of the most significant modifiable risk factors and increased awareness of this disease. The impact of PPD on the mother-child relationship has consequences that can affect the entire family. Family members and partners should be supportive and share responsibilities with mothers during pregnancy and the postpartum period, as this reflects positively on the mother’s mental health, thereby playing an important role in decreasing the prevalence of PPD. In addition, we recommend early screening programs and continuous follow-up to monitor the mother’s mental health and facilitate early intervention and support from competent authorities, if needed. Moreover, close follow-up of patients with an emphasis on other types of psychological diseases is important.
